# Bridge formation method for endoscopic submucosal dissection of early gastric neoplasms along the lesser curvature at the gastric angle

**DOI:** 10.1055/a-2774-3306

**Published:** 2026-01-22

**Authors:** Junhao Liu, Weixing Yang, Xuanli Wang, Chunhai Fu, Zhongqiong Wang, Xiaowei Tang

**Affiliations:** 1 556508 Department of Gastroenterology, The Affiliated Hospital of Southwest Medical University, Luzhou, China

Endoscopic submucosal dissection (ESD) is an effective method for the en bloc resection of early gastrointestinal neoplasms. For gastric lesions, pocket or modified pocket ESD is typically recommended when tunnel creation is feasible 1 2 . However, large gastric lesions present challenges in determining when to stop submucosal dissection, as the resection margins are not visible within the pocket.

To enhance traction and precision, several modified ESD techniques have been developed in colorectal practice, such as the “tunnel-and-bridge” method and the bridge formation method 3 4 5 . These approaches involve creating submucosal tunnels while preserving mucosal “bridges” for countertraction, improving dissection efficiency and increasing en bloc and R0 resection rates. However, these “bridge ESD” techniques have not yet been reported to treating gastric lesions.

**Fig. 1:**
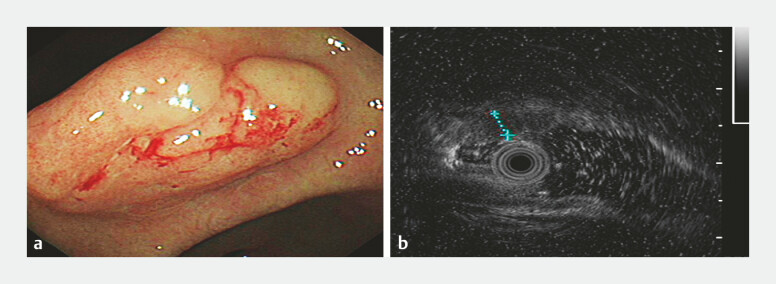
Endoscopic and ultrasound findings of a gastric lesion at the gastric angle. **a** An endoscopic image showing a 3.0 cm × 2.2 cm elevated lesion with a 0-IIa + IIc appearance and surface congestion. **b** An endoscopic ultrasound (EUS) image depicting thickening of the mucosal muscular layer, with reduced echogenicity. The lesion has a diameter of approximately 0.3 cm at the deepest point. The submucosal layer remains continuous, and the gastric wall layers are clearly defined.

**Fig. 2:**
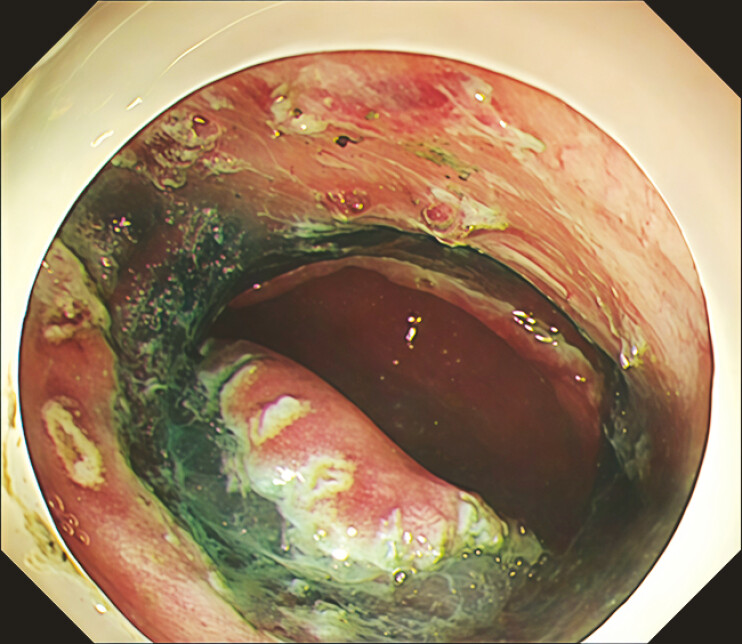
An endoscopic image showing the formation of a mucosal bridge during the ESD procedure. ESD, endoscopic submucosal dissection.

**Fig. 3:**
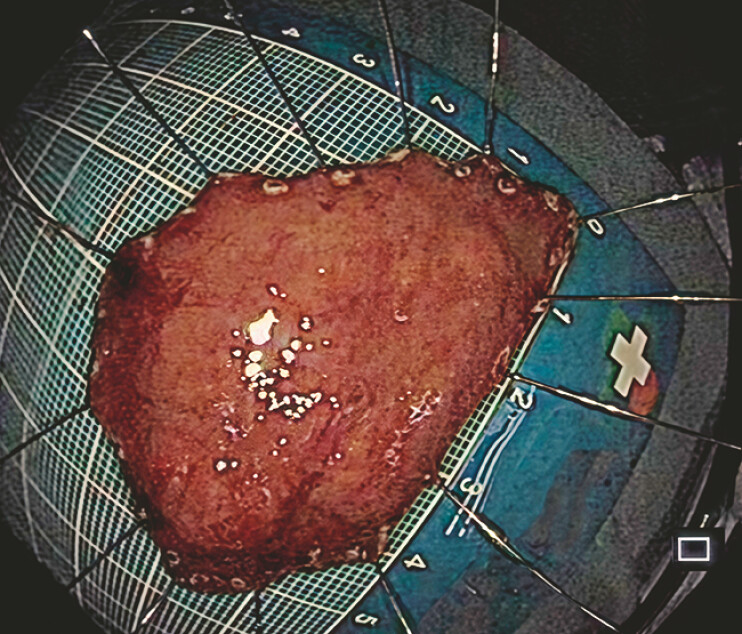
The resected specimen following bridge ESD. ESD, endoscopic submucosal dissection.

**Fig. 4:**
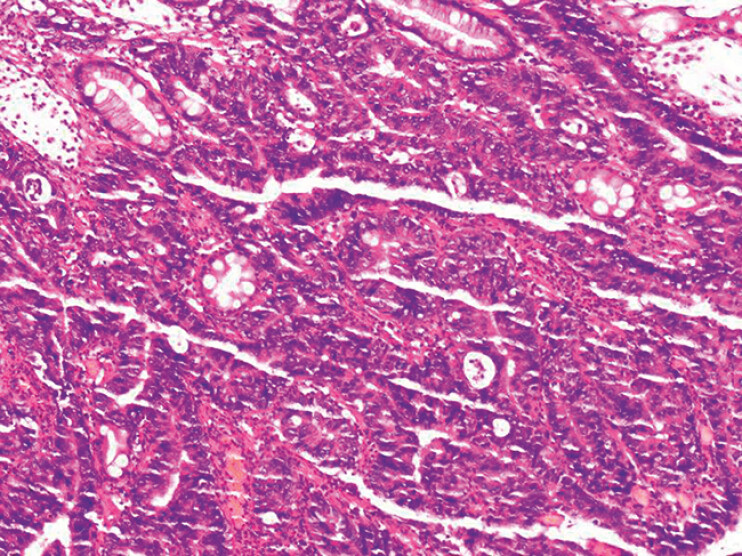
A histopathological image of the resected lesion showing high-grade intraepithelial neoplasia with focal intramucosal carcinoma.

Here, we presented a case where bridge ESD was used to resect a gastric tumor ( Video 1 ). A 68-year-old woman had a 3.0 cm × 2.2 cm elevated lesion on the lesser curvature at the gastric angle, where sharp angulation and paradoxical endoscope movement pose unique challenges ( Fig. 1 ). ESD was performed using the bridging technique. Mucosal incisions were made from the anal and oral sides, followed by submucosal dissection from the oral side. A tunnel connecting both sides was created beneath the lesion, forming a “bridge” ( Fig. 2 ). The intact mucosal bridge provided direct tissue traction, facilitating the dissection. Finally, the “pillars” on both sides of the bridge were removed, achieving a complete (R0) resection ( Fig. 3 ). Histopathology confirmed high-grade dysplasia with intramucosal carcinoma ( Fig. 4 ). No complications occurred, and the patient recovered uneventfully.

Endoscopic submucosal dissection using the bridge formation method for a gastric angular lesion.Video 1

In this case, bridge ESD effectively overcomes the limitations of pocket ESD by creating a mucosal bridge that provides natural countertraction, enhancing the resection of large gastric lesions and improving both visibility and precision. This technique demonstrates high efficacy in managing large and complex gastric lesions, reducing procedural challenges, and may serve as a vital tool in treating difficult gastric conditions.

Endoscopy_UCTN_Code_TTT_1AO_2AG_3AD
